# Cognitive primitives of the insect brain

**DOI:** 10.1016/j.tics.2025.11.013

**Published:** 2025-12-05

**Authors:** Scott Waddell, Annie Park

**Affiliations:** 1 Centre for Neural Circuits and Behaviour, University of Oxford, Oxford, OX1 3TA, UK

## Abstract

Understanding the mechanistic basis of human cognition is likely to benefit from investigating how it emerged through evolution. We propose that identifying and investigating fundamental brain functions, or cognitive primitives, common between humans and ‘lower’ animals, such as insects, will reveal conserved cellular and molecular operations underlying cognition.

## Origins of cognition

Human intelligence appears to be exceptional, yet it must have arisen through evolution as a consequence of the unique selective pressures faced by hominids. Many widely accepted examples of cognitive function – such as language, reasoning, decision-making, and planning – have historically been framed to idealize uniquely human abilities. While these constructs have critically shaped the field, they were often developed with the implicit goal of differentiating humans from other animals. Thus, it is hardly surprising that some still contend that cognition cannot be applied to so-called ‘lower’ organisms. To challenge this assertion, we argue that there is value in deconstructing complex cognitive abilities into their basic operations and reframing the discussion around how these abilities arise from simpler, fundamental molecular and circuit-level mechanisms [1].

Towards this goal, we coin the term ‘cognitive primitive’ to encourage consideration of elementary cognitive processes and to define them in a manner that allows quantitative experimental testing in animal models traditionally viewed as lacking cognition. Although we describe examples from *Drosophila melanogaster* and other insects, cognitive primitives should exist in some form in more primordial species.

## Deliberation before action

All natural environments are dynamic and ambiguous. As a result, animals regularly need to evaluate perceptually cryptic stimuli – most of which are not explicitly reinforced – and adapt their behavior to survive. Ambiguity exists on both perceptual and predicted value levels, which compels animals to carefully evaluate stimuli before committing to a behavioral response. Deliberation requires time, increases with the degree of ambiguity, and contrasts with the classical stimulus–response model, which is typically ascribed to many non-human animals and considered non-cognitive. We propose deliberation as a cognitive primitive, measurable as the latency between sensory input and behavioral output. Although processes such as attention, perception, memory, decision-making, and reasoning all contribute to deliberation, deliberation provides a measurable, broadly applicable framework for cross-species comparisons, and it can be applied to organisms like drosophila with less contention than traditional cognitive terms.

Evidence for insect deliberation comes from studies examining neuronal mechanisms of decision-making that do not happen immediately. Like mammals, insects show increased decision latency in both perceptual and value-based tasks as task difficulty increases. In drosophila, perceptual discrimination between different concentrations of the same odor relies on evidence accumulation within the olfactory αβ Kenyon cells of the mushroom bodies, which adjust their firing rate such that they are slower to fire as odor concentrations become more similar and thus more difficult to distinguish [2]. Female drosophila also carefully evaluate substrate quality for egg-laying. A neural correlate of deliberation before egg-laying is evident in six oviposition descending neurons, whose activity is driven by a rise-to-threshold integration over a timescale of minutes that encodes the relative quality of the substrate [3]. Gradual commitment to a decision is also reflected in asymmetric calcium activity in the neurons forming the inferior posterior slope of the fly brain, which can predict turning direction up to ten seconds before the fly executes a turn [4]. Other insects show analogous evidence of deliberation. For example, individual honeybees (*Apis mellifera*) opt out of engaging in especially challenging perceptual discrimination tasks, indicating that they have developed an understanding of relative difficulty [5].

Together, these findings suggest that insect decision-making relies on temporally extended evidence accumulation and evaluation mechanisms, rather than immediate reflexive responses.

## Adaptive active sampling requires working memory

In dynamic and uncertain environments, animals must flexibly alternate between exploring to gather information and exploiting resources. We propose that this strategy, known as spontaneous alternation or active sampling, also represents a cognitive primitive. It can be directly measured through behavior and computationally modelled to reveal underlying neuronal/cognitive processes. The ‘infotaxis’ framework [6] describes how animals choose actions that maximize the expected rate of information gain, based on moment-to-moment sensory input. According to this perspective, behavior actively shapes perception and internal representations, allowing animals to guide their own reinforcement through interaction with the environment.

In drosophila, spontaneous alternation and active sampling reveals search strategies consistent with infotaxis. During olfactory navigation, flies move upwind and track along the edges of an odor plume. In turbulent odor fields, walking flies periodically pause and reorient to maximize information gain, a pattern requiring short-term integration of sensory history [7]. Weaving in and out of an odor plume relies on a vector-based computation in the central complex, where the plume boundary is remembered and used as a dynamic landmark [8]. Active sampling is not limited to olfactory contexts: during exploration of a novel environment in a Y-maze, flies exhibit spontaneous alternation in turning behavior, which habituates over time.

Active sampling of the environment in an adaptive manner suggests that there is an internal model of the world that is being updated based on the actions of the individual. We argue that active sensing can be considered a form of working memory.

## Learning without immediate reinforcement

A third cognitive primitive is the ability to learn without immediate reinforcement. One of the most evolutionarily ancient forms of learning likely stems from the need to associate sensory cues (e.g., smell and taste) during feeding with the delayed post-ingestive consequences of food consumption [9]. As animals evolved to consume more diverse diets, these mechanisms likely adapted to have greater flexibility due to the complexity and variability of post-ingestive feedback (e.g., nutrient-specific value or toxicity), laying the groundwork for more abstract forms of learning beyond direct cue–reward pairings. Insects are capable of learning using post-ingestive feedback as they selectively remember olfactory associations with nutritive sugars for the long term, and update their behavior depending on the presence of secondary post-ingestive reinforcers such as toxins (e.g., [10]). They can also transfer a previously learned nutrient-predictive value from one odor to another new odor during second-order conditioning. Non-explicit reinforcement can also arise through social learning, such as when flies and many other insects – *Venturia canescens* pre-imaginal prey conditioning to a non-typical host, *Schistocerca americana* conditioned taste avoidance, and *Lasioglossum zephyrum* suppression of mating between related individuals by odorant similarity – acquire preferences by observing the behavior of others: for example, predator avoidance, oviposition site choice, or mate selection [9,11].

## Generalization is required to survive in a dynamic environment

An important extension of learning without explicit reinforcement is the ability to generalize learned information across differing contexts: a necessity in natural environments where reward-predictive cues are rarely present in the same exact combinations. Flies can extract general rules from learned experiences and apply them to new situations, such as in visual pattern learning, during which associations persist even when visual stimuli are displaced or presented in different colored backgrounds, indicating that flies have the capacity to learn what a pattern is and apply the rule irrespective of position, size, and background color [12]. Honeybees and bumblebees (*Bombus terrestris*) can also learn relational rules such as same/different, above/below, or larger/smaller and apply them to completely novel visual stimuli [5]. Generalization comes in far more complex forms (e.g., singing a tune or playing it on the piano), but we also consider sensory generalization to be a fourth cognitive primitive.

## Concluding remarks

A mechanistic understanding of cognition benefits from identifying conserved computational functions across species at the molecular and cellular levels. The four cognitive primitives we outline here may be implemented by homologous circuit motifs and signaling pathways from invertebrates to mammals. For instance, conserved neuromodulatory systems that represent the current need of the animal bias the behavior they tend to execute, and are likely to influence all of the aforementioned cognitive primitives (e.g., hungry animals may have reduced deliberation time, spend more time exploiting their environments, and show an increased ability to generalize) [13]. Even in the mollusc *Aplysia californica*, operant reward learning reveals a role for dopamine in reinforcing and regularizing feeding actions (biting) [14], suggesting deep conservation of neuromodulatory principles underlying goal-directed behaviors. More complex cognitive functions in humans may therefore emerge from the expansion and diversification of existing cognitive principles, rather than entirely novel mechanisms. Evolutionary expansion and integration of new neural cell types provides a means to broaden the repertoire of physiological computations and enable more sophisticated cognitive processing to emerge.

## References

[1] Cisek P (2019). Resynthesizing behavior through phylogenetic refinement. Atten Percept Psychophys.

[2] Groschner LN (2018). Dendritic integration of sensory evidence in perceptual decision-making. Cell.

[3] Vijayan V (2023). A rise-to-threshold process for a relative-value decision. Nature.

[4] Brezovec LE (2023). Neural correlates of future volitional action in Drosophila. bioRxiv.

[5] Avarguès-Weber A, Giurfa M (2013). Conceptual learning by miniature brains. Proc R Soc B Biol Sci.

[6] Vergassola M (2007). ‘Infotaxis’ as a strategy for searching without gradients. Nature.

[7] Pang R (2018). History dependence in insect flight decisions during odor tracking. PLoS Comput Biol.

[8] Siliciano AF (2025). A vector-based strategy for olfactory navigation in Drosophila. bioRxiv.

[9] Papaj DR, Lewis AC (1993). Insect Learning: Ecological and Evolutionary Perspectives.

[10] Wright GA (2010). Parallel reinforcement pathways for conditioned food aversions in the honeybee. Curr Biol.

[11] Perry CJ (2017). The frontiers of insect cognition. Curr Opin Behav Sci.

[12] Tang S (2004). Visual pattern recognition in Drosophila is invariant for retinal position. Science.

[13] Senapati B (2019). A neural mechanism for deprivation state-specific expression of relevant memories in Drosophila. Nat Neurosci.

[14] Brembs B (2002). Operant reward learning in Aplysia: neuronal correlates and mechanisms. Science.

